# The Efficiency of Rehabilitation Therapy in Patients Diagnosed with Neurogenic Bladder: A Systematic Review

**DOI:** 10.3390/medicina60071152

**Published:** 2024-07-17

**Authors:** Adina Ionelia Manaila, Nadinne Alexandra Roman, Ionut Cristian Cozmin Baseanu, Diana Minzatanu, Vlad Ionut Tuchel, Elena Bianca Basalic, Roxana Steliana Miclaus

**Affiliations:** 1 Department of Fundamental, Preventive, and Clinical Disciplines, Faculty of Medicine, Transilvania University of Brasov, 500036 Brasov, Romania; 2 Neurorehabilitation Department, Clinical Hospital of Psychiatry and Neurology, 500036 Brasov, Romania

**Keywords:** neurogenic bladder, physical therapy, rehabilitation, stroke, multiple sclerosis, electrophysical agents, spinal cord injury, pelvic floor

## Abstract

Considerable research efforts have been directed towards investigating neurogenic bladder dysfunction over the preceding decade. This condition stands as the most prevalent and incapacitating pelvic floor disorder amidst patients afflicted with specific upper motor neuron syndromes, including multiple sclerosis, stroke, and spinal cord injury. The current study aims to bring up-to-date findings on rehabilitation methods for treating neurogenic bladder. The Web of Science database (MEDLINE, PsychINFO, EMBASE, CENTRAL, ISRCTN, and ICTRP) was screened for randomized controlled studies and clinical studies using combinations of keywords including “neurogenic bladder”, “stroke”, “multiple sclerosis”, and “spinal cord injury”. The PEDro scale was used to assess the quality of the articles included in this study. After a thorough examination, eleven articles met the criteria for inclusion in our research. The outcome measures showed a variety of forms of electrostimulation that can be combined with or without PFMT. These interventions significantly enhance health-related quality of life, as evidenced by various assessment methods. The physical approach constitutes an effective therapeutic method that can reduce the severity of urinary incontinence.

## 1. Introduction

The physiological process of micturition relies on the proper coordination of the urinary bladder and urethral sphincters [1]. Disruption of voluntary control of micturition due to nervous system injuries or illnesses in adults can lead to bladder overactivity and urge incontinence [2]. Neurogenic bladder refers to detrusor muscle overactivity resulting from dysfunctions in the bladder’s smooth muscle or innervation [3,4]. Overactive bladder syndrome (OAB) is characterized by abrupt urges to urinate, frequent daytime voiding, and nocturia, with or without incontinence [3,5].

### 1.1. Stroke

Stroke episodes manifest as abrupt clinical indications of cerebral function impairments, usually stemming from vascular issues. They can result in cognitive and sensorimotor alterations [6]. Following a stroke, urinary incontinence (UI) frequently occurs and serves as a prognostic factor for unfavorable outcomes [7]. About 80% of UI cases affect the lower urinary tract [7]. Precise diagnosis and management of bladder dysfunction are essential for enhancing patient well-being and mitigating long-term complications [8].

### 1.2. Multiple Sclerosis (MS)

Multiple sclerosis (MS) is a chronic neurological disorder that significantly impacts a patient’s quality of life [9]. Between 19 and 80% of individuals with MS experience urinary incontinence, which may be a result of neurogenic bladder or lower urinary tract dysfunction [10]. Although it is not life-threatening, MS can lead to negative emotions such as embarrassment, shame, reduced self-esteem, sexual dysfunction, isolation, and limitations in social activities [9].

### 1.3. Spinal Cord Injuries (SCI)

The annual global incidence of spinal cord injuries leading to neurogenic bladder dysfunction ranges between 12 and over 65 cases per million population [11]. This condition commonly occurs in individuals with spinal cord injuries, affecting about 95% of those with injuries above the sacral region [11,12]. It often leads to challenges such as urinary retention, incontinence, UTIs, renal complications, and reduced quality of life [11,12]. Management involves strategies such as timed voiding, intermittent catheterization, indwelling catheterization, and the use of medications for overactive bladder [13,14]. However, these strategies do not address the reorganization of spinal reflexes contributing to detrusor hyperreflexia and detrusor–sphincter dyssynergia (DSD) [15].

### 1.4. Pharmacological Therapy

Antimuscarinic drugs, beta-3 adrenergic receptor agonists, and cannabinoids are used to alleviate neurogenic bladder dysfunction symptoms [16]. These medications help increase bladder capacity, reduce urinary incontinence episodes, and manage lower urinary tract symptoms [16,17]. Also, alpha-blockers can minimize bladder outlet resistance and detrusor pressure [16].

### 1.5. Catheterization

Catheterization is a minimally invasive treatment option [16]. Intermittent self-catheterization or catheterization performed by a third party is preferred for neuro-urological patients having difficulty emptying their bladders [16]. Adequate instruction in self-catheterization is crucial to minimize UTI risk. Ideally, bladder volume at catheterization should be 400–500 mL [16].

### 1.6. Botulinum Toxin A

Botulinum toxin A is a minimally invasive treatment option for DSD, administered through sphincter injections at a tailored dosage [3,16,18]. Its mechanism involves inducing long-lasting yet reversible chemical denervation, typically around nine months [16]. Clinical studies have confirmed its effectiveness in managing neuro-urological disorders associated with conditions like MS, SCI, and Parkinson’s disease [16,18,19].

### 1.7. Rehabilitation Treatment

The goal is to reduce urinary urgency or incontinence and ensure proper bladder emptying [10]. This involves finding the right balance in managing symptoms related to neurogenic detrusor overactivity and bladder retention [10]. Physical approaches for treating neurogenic bladder include assisted voiding techniques (such as the Credé maneuver, Valsalva maneuver, and triggered reflex voiding), pelvic floor muscle training (PFMT), and electric stimulation [16]. Electric stimulation is a therapeutic intervention with minimal adverse effects, and it has been studied for lower urinary tract symptoms (LUTS) [16]. The most common are high-intensity electromagnetic stimulation [20], repetitive transcranial magnetic stimulation (rTMS) [21], intravesical electrical stimulation (IVES) [22], transcutaneous tibial nerve stimulation (TENS) [6], posterior tibial nerve stimulation (PTNS) [6,21,23,24,25,26,27], interferential electrical stimulation (IMFC ES) [28], parasacral nerve electrostimulation (PSES) [6], pelvic floor muscle training (PFMT) with electromyographic (EMG) biofeedback [26,29], neuromuscular electrical stimulation (NMES) [29], percutaneous tibial nerve stimulation (PtNS) [30,31], and dorsal genital nerve stimulation (DGNS) [16]. Other approaches include lifestyle interventions [10,16], anti-incontinence devices [10], and general exercises using virtual reality (VR) [32].

Bladder dysfunction is a common issue for individuals with stroke, MS, and SCI. However, effective therapeutic strategies have not been thoroughly explored, especially in the context of rehabilitation. Although there is growing interest in physical approaches, there is still a notable gap in the literature regarding identifying effective rehabilitation methods and interventions for neurological bladder symptoms.

This paper aims to systematically review the effectiveness of various physical therapy interventions, such as pelvic floor muscle training (PFMT) and electrostimulation, in managing neurogenic bladder dysfunction in patients with neurological conditions. The primary focus is identifying the efficiency of noninvasive treatments in reducing urinary incontinence and improving patients’ quality of life. Additionally, this study seeks to identify and discuss the assessment methods used in the reviewed studies to evaluate neurogenic bladder issues, ensuring a comprehensive understanding of treatment effectiveness and patient outcomes.

## 2. Materials and Methods

This study follows and satisfies the essential components outlined in the PRISMA (preferred reporting items for systematic reviews and meta-analysis); the registration statement for PRISMA checklist can be found in Supplementary File [33].

### 2.1. Search Strategy

A search was started on the Web of Science Core collection database. We selected the following databases: MEDLINE, PsychINFO, EMBASE, CENTRAL, CENTRAL, ISRCTN, and ICTRP from the WOS platform due to their broad coverage of medical specialties, especially in the rehabilitation field. The search was performed on 30 April 2024 and was completed on 3 May 2024. The refinement of the studies was conducted in the period 4 May to 15 May 2024. The search period spanned 10 years and 4 months (1 January 2013–30 April 2024). Our search strategy included studies published up to 1 January 2013 to ensure a focus on the most recent evidence available at the time of study initiation. This cut-off date captured the most relevant and contemporary literature while allowing sufficient time for comprehensive data collection and analysis. We searched for articles related to the topic. We used combinations of keywords including “neurogenic bladder” AND “physical therapy”—35 results, “neurogenic bladder” AND “electrophysical agents”—1 result, “neurogenic bladder” AND “rehabilitation”—215, “neurogenic bladder” AND “electrostimulation”—12 results, “neurogenic bladder” AND “stroke”—56 results, “neurogenic bladder” AND “multiple sclerosis”—297 results, “neurogenic bladder” AND “spinal cord injury”—872 results, “neurogenic bladder” AND “interferential currents”—2 results, and “neurogenic bladder” AND “transcutaneous electrical nerve stimulation”—22 results. The filter used was “Article”. In total, we obtained 1512 results. The search was not restricted by language but was performed using keywords in English. One of the included studies in our review had the title and the abstract in English and the full text in French. We included a relevant study published in languages other than English after a thorough translation process was employed for this non-English article to meet the inclusion criteria.

The eligibility criteria identified (1) the population, (2) interventions, (3) comparisons, (4) outcomes, and (5) study designs. The PICOS strategy was followed during the initial phase [34]. The included (1) patients were subjects diagnosed with stroke, MS, or SCI and included both men and women. The (2) intervention was rehabilitation using physical therapy. The (3) comparison consisted of the intervention on two or more groups of patients as appropriate, in which the following were analyzed: PFMT versus an electrostimulation technique (7 studies), an electrostimulation technique versus pharmacological therapy (1 study), or two different electrostimulation techniques (3 studies). The (4) outcomes include bladder function, voluntary detrusor contractions, urinary leakage volume, daily urinary incontinence episodes, and pelvic floor muscle strengthening. The (5) study designs consisted of 10 randomized controlled trials (RCTs) and one clinical controlled trial (CT) that used IVES, PTNS, PtNS, PSES, PFMT, and NMES with and without EMG biofeedback.

Review or meta-analysis papers, case reports, validation, and feasibility studies were excluded. Irrelevant to the research were the studies on patients with neurogenic bladder other than those caused by stroke, MS, SCI, or other neurological conditions, which were excluded from the paper.

### 2.2. Selection Process

After collecting all the results, we removed any duplicate entries. Four independent reviewers (A.I.M., I.C.C.B., D.M., E.B.B.) carried out the selection process.

**Figure 1:**
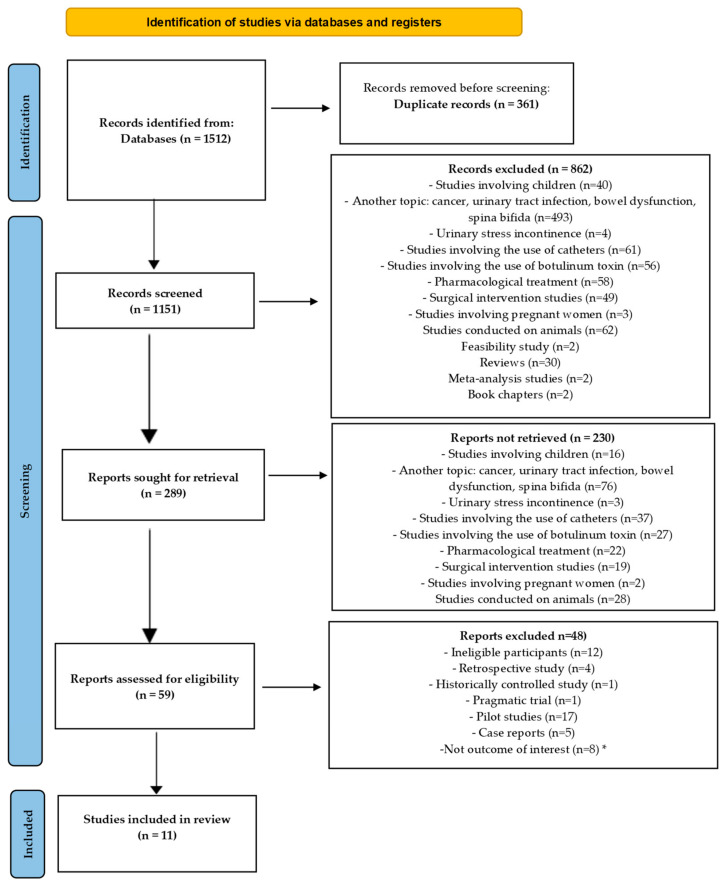
PRISMA diagram flow. * = studies focused on assessing activities of daily living in patients with NB (n = 4), studies aimed at validating the SF-Qualiveen questionnaire (n = 3), and a study (n = 1) analyzing physical factors that may cause detrusor contraction.

A total of 1512 articles were generated in the database searches. Duplicates were eliminated, excluding 361 articles assessed by title and abstract according to the PICO model [34]. The next step involved reviewing the studies based on their titles (862 articles were excluded) and evaluating their abstracts (230 articles were excluded). Finally, we thoroughly reviewed the full texts and selected the eligible studies for inclusion in our review. Fifty-nine studies were thoroughly reviewed according to the eligibility criteria. Forty-eight studies were reviewed, and twelve were excluded due to ineligible participants. Four retrospective studies were excluded. Additionally, 24 studies were excluded because of ineligible designs, which included 1 historically controlled study, 1 pragmatic trial, 17 pilot studies, and 5 case reports. The last category included eight studies that were not relevant to our field of interest: four studies focused on assessing activities of daily living in patients with neurogenic bladder, three studies aimed at validating the SF-Qualiveen questionnaire, and one study analyzed physical factors that may cause detrusor contraction. Ultimately, 11 articles were included, as shown in Figure 1.

## 3. Results

### 3.1. Characteristics of Included Studies

In our paper, we included 11 studies that analyzed the effects of different forms of physical therapy on 364 patients diagnosed with neurogenic bladder. Thus, 24 patients (men) were diagnosed with stroke, 229 patients (men and women) were diagnosed with MS, and 155 patients (119 men and 36 women) were diagnosed with SCI.

The studies deemed eligible were published between 2014 and 2023. We evaluated the quality of the selected studies that met the inclusion criteria using an assessment tool called the PEDro scale (Table 1). This tool can help readers understand how much confidence they should place in the findings of a study [35]. The PEDro scale consists of 11 criteria for assessing randomized controlled trials’ external and internal validity [35].

It is important to note that Item 1 is not scored. The PEDro scale assesses internal validity and statistical reporting [35]. Items 2 to 9 cover aspects such as allocation, blinding, completeness of follow-up, and intention-to-treat analysis, while items 10 to 11 focus on statistical reporting, including between-group comparisons, mean, and variability data [35]. The total PEDro score is calculated by adding up the points assigned to items 2 through 11, resulting in a score ranging from 0 to 10 [35]. Two items pertain to blinding of therapists and participants, which may be challenging to achieve in physical activity interventions [35].

In the 11 presented studies, a wide range of therapies was used, as follows: three studies with TTNS [12,23,29], four studies with PFMT [25,36,38,39], one study with unguided PFMT [38], one study in which the patients performed a set of activities at home [39], three studies with PTNS [24,25,26], one study with PFMT and EMG biofeedback and sham NMES [29], one study with PFMT and EMG biofeedback and intravaginal NMES [29], one study with PFMT with EMG biofeedback and TTNS [29], one study with PFMT and EMG biofeedback using a manometric anal pelvic floor probe [26], one study with muscle-stretching training [24], two studies with PtNS [37], one study with Solifenacin Succinate [31], and two studies with PFMT and IVES [36,39]. The results obtained can be seen in Table 2.

### 3.2. The Quality of the Studies

Our research has included studies on managing pelvic floor dysfunction that have yet to use standardized protocols and have included a small number of heterogeneous patients. These studies have primarily relied on a conservative, noninvasive approach that includes a variety of physical treatment forms over different time durations and is focused on reducing urinary incontinence symptoms.

### 3.3. Assessment Methods

Assessment tools, such as assessment scales, have shown subjective improvements in outcomes following specific treatments. The international consultation on incontinence questionnaire short form (ICIQ-SF) [23,29,36,38], the over-active bladder awareness tool—8-item (OAB-V8) [23,25,29,38], the bladder diary for three days [23,24,29,31,37,38], the Barthel index [24], the SF-Qualiveen [26,29], and the health-related quality of life (HRQOL) [39] have demonstrated significant improvements after interventions like pelvic floor muscle training (PFMT) or tibial nerve stimulation. Objective tools for patient evaluation included abdominal ultrasound [25], urodynamic studies such as pressure-flow study [36], cystometry [12,23,25,29,36], and, importantly, pelvic floor muscle assessment [31,36].

These tools are valuable for measuring treatment effectiveness and patient-reported outcomes, thereby facilitating a comprehensive understanding of the impact of these interventions on urinary incontinence and overall quality of life.

### 3.4. Types of Noninvasive Interventions

Kegel proposed PFMT in 1948 through repeated exercises aimed at “the functional restoration of the perineal muscles” [40]. In the study, Bø outlined three theories regarding PFMT: one focused on behavioral aspects, aiming to teach conscious pre-contraction of the pelvic floor muscles before and during rises in abdominal pressure to prevent leaks, and two centered on altering neuromuscular function and morphology [41].

Several studies explored PFMT either independently, with or without supervision from a physiotherapist [36,38], or in combination with other approaches or devices, such as intravaginal NMES [26,36,39], EMG biofeedback [26,29], TTNS [29], or PTNS [25] in patients diagnosed with MS or SCI. For stroke patients, sessions involving stretching of the lower limb muscles were employed as part of the treatment regimen [24]. From this standpoint, gathering data regarding the advantages of long-term protocols for PFMT would be valuable.

**Figure 2:**
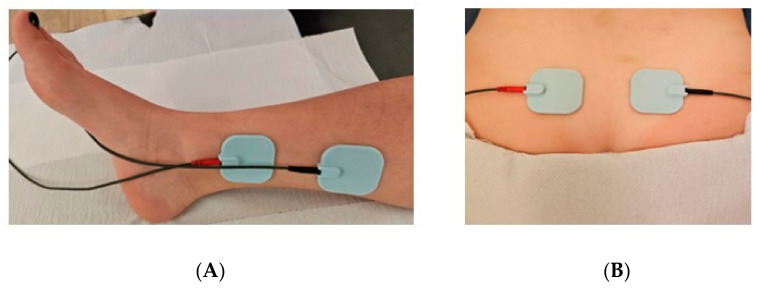
Application of electrodes in tibial nerve electrostimulation techniques (**A**) and parasacral electrostimulation (**B**) [6].

Tibial nerve stimulation has emerged as a conservative, alternative treatment option to reduce lower urinary tract symptoms [23]. A variety of forms of tibial nerve electrostimulation have been observed in our research, such as IVES [21], TENS [6], PTNS [17,25,26,31], and PtNS [30,31]. It is primarily used for patients with overactive bladder syndrome who do not respond to behavioral or pharmacological treatment [12,23]. Additionally, it has proven to be a valuable therapy for neurogenic detrusor overactivity, chronic pelvic pain syndrome, and fecal incontinence [23]. McGuire et al. successfully initiated this neuromodulation method as an electroacupuncture technique to stimulate the tibial nerve near the medial malleolus for the first time in 1983 [23,42]. Later, Ramírez-García et al. demonstrated that using two transcutaneous applications is safer and easier to apply than the needle-based percutaneous approach while showing noninferiority in its clinical efficacy [23,43]. In their study, Albello A. et al. [44] mentioned that TENS presents several similarities with Asian acupuncture techniques; however, it is perceived as a peripheral, minimally invasive form of sacral neuromodulation. Sacral neuromodulation and tibial nerve stimulation are based on the following theory: stimulation of the S3 nerve root (the S3 root consists of afferent and efferent fibers. The position of the electrodes can be seen in Figure 2. And it is hypothesized that neuromodulation reduces sensory input and inhibits motor reflex circuits in the pontine micturition center at a relatively low amplitude and modulates the neural activity of several pathways involved in bladder control [23,44].

## 4. Discussion

The main goal of this comprehensive review was to assess the effectiveness of various physiotherapy treatments, including pelvic floor muscle training (PFMT) and electrostimulation, compared to placebo (sham stimulation), drug therapy, or unguided PFMT. The analysis of 11 studies provided compelling evidence supporting the positive impact of noninvasive treatment options for managing neurogenic bladder in patients with neurological conditions. Specifically, the review included seven studies involving patients diagnosed with MS, one study with individuals diagnosed with stroke, and three studies with patients diagnosed with SCI.

The impact of urinary incontinence symptoms on neurological patients can be quite severe, affecting their quality of life in various ways [45]. These symptoms can lead to financial difficulties, decreased self-esteem, a heightened risk of falls, and a greater likelihood of developing urinary tract infections. A significant amount of research has been conducted to identify the most effective approaches to managing neurogenic bladder dysfunction. For example, Santiago J.E. et al. [2] reviewed various therapeutic options, including invasive, minimally invasive, and noninvasive methods. Additionally, researchers such as Sapouna V. et al. [9], Vecchio M. et al. [10], Sparaco M. et al. [40], and Zecca C. et al. [46] have focused on investigating the benefits of noninvasive methods for patients with urinary dysfunction caused by conditions like multiple sclerosis. Some of these noninvasive methods are also discussed in our study.

The management of neurogenic bladder through rehabilitation methods is the focus of substantial interest within the medical community. This is supported by the extensive body of evidence from randomized clinical trials conducted thus far [12,23,24,25,26,29,31,36,37,38,39,47,48,49], with additional ongoing studies such as the research led by Dandan H.B.A et al. [50] and Birkhäuseret V. et al. [51]. Furthermore, there are forthcoming studies indicated by the publication of protocols for RCT studies by Candido et al. [6], Cakir et al. [21], and Xu L. et al. [52].

In addition to neurogenic bladder, considerable attention has been directed towards treating urinary dysfunction stemming from etiologies other than neurogenic bladder. These conditions may encompass stress urinary incontinence, pelvic organ prolapse, and various related issues.

The reviewed studies utilized various assessment methods to examine neurogenic bladder issues and the effectiveness of different interventions. Subjective tools such as the ICIQ-SF [23,29,36,38] and the OAB-V8 [23,25,29,38] were commonly used, and they showed significant improvements after the interventions. Additionally, bladder diaries [23,24,29,31,37,38], maintained over three days, provided valuable insights into the frequency and severity of incontinence episodes. Objective evaluation methods included abdominal ultrasounds [25], pressure-flow studies [36], and cystometry [12,23,25,29,36], which offered precise measurements of bladder function and dysfunction.

Pelvic floor muscle assessment, conducted through digital vaginal and rectal palpation [37], as well as EMG biofeedback [36], was crucial in identifying muscle strength and functionality. Together, these tools facilitated a comprehensive evaluation of treatment outcomes, highlighting improvements in urinary symptoms and overall quality of life. Integrating these assessment methods ensures a holistic approach to managing neurogenic bladder dysfunction, emphasizing the importance of subjective patient-reported outcomes and objective clinical measurements to guide treatment decisions and enhance patient care.

Silantyeva E. et al. [53] conducted a study to explore the impact of high-intensity-focused electromagnetic (HIFEM) therapy on postpartum women. HIFEM involves using targeted electromagnetic energy to elicit supramaximal contractions in the pelvic floor muscles [53]. This process entails using a device comprising a generator linked to a chair housing the stimulation coil. The coil emits a focused magnetic field with intensities reaching up to 2.5 Tesla, capable of triggering muscle contractions at depths of up to 10 cm. Patients were seated in a chair for each 28 min session during the study. The stimulation strength was adjusted on a 0 to 100% scale (equivalent to 0 to 2.5 Tesla) based on patient feedback up to the maximum tolerable threshold, wherein patients experienced muscular contractions without pain or discomfort [53]. Two successive treatments were administered, with a minimum 48 h gap between sessions, to mitigate muscle fatigue, as the contractions induced are significantly more intense than those achieved through voluntary exercise [53]. Patients treated with HIFEM technology exhibited notably more pronounced improvement in EMG values, signifying the significant effectiveness of BTL EMSELLA technology in restoring pelvic floor muscle strength [53]. The potential of this therapeutic approach warrants investigation in neurologically impaired patients with neurogenic bladder, as it is noninvasive and appears to be free of adverse effects, at least in the early stages of research.

The abbreviation TECAR (transfer electrical, capacitive and resistive) was initially developed in Italy. TECAR is a radiofrequency (RF) therapy that utilizes high-frequency monopolar capacitive–resistive radiofrequency waves [54]. High-power TECAR therapy devices induce an electric field within the tissue, leading to the movement of charged particles and the generation of heat [55]. The application of electromagnetic energy within the body initiates biological and physiological responses. As the body is a secondary conductor, it comprises a substantial amount of water with numerous dissolved ions. Applying electromagnetic energy accelerates metabolic reactions at various levels for therapeutic purposes. TECAR therapy utilizes energy ranging from 0.8 to 1.2 MHz for treatment. The mechanism of action involves using high-frequency electricity to create heat in body tissues due to the resistance these tissues offer to the electric current [55].

A study by Elhosary E.A. et al. investigated the effects of TECAR therapy in women diagnosed with stress urinary incontinence. The technique involved applying a sterile electrode with an intravaginal lubricating gel for 20 min per session, at a power range of 1–5%, at a subthermal level. The treatment consisted of 12 sessions of RF 3 times a week for 4 weeks. This study demonstrated that this therapy provides benefits in alleviating treated patients’ symptoms. Research into the effects of TECAR therapy has expanded, including a study by Franić D. et al., which examined the impact of this therapy in patients diagnosed with overactive bladder (OAB) with or without urinary urge incontinence (UUI) [54]. Electrodes were situated on the lower abdomen in the bladder area (active electrode) and the lumbar spine region (indifferent electrode) [54]. A capacitive probe in the “free treatment”/“power control” program, where high-frequency electricity was used, was applied to the bladder for 20 min at a frequency of 1.0 MHz and with energy limited to that which could produce a maximum temperature of 41 °C [54]. The RF application protocol in this study was 1× weekly/20 min/4 weeks [54]. Similarly, statistically significant results were identified in this pilot study two weeks after the start of treatment. Further research on TECAR therapy should be pursued, as it represents an alternative, noninvasive physical treatment for OAB symptoms.

Rutkowska A. et al. [32] and Elliott V. et al. [56] investigated the effects of exercise via virtual reality in patients diagnosed with urinary dysfunctions, highlighting the emphasis on finding new, interactive, noninvasive, side-effect-free treatment methods that appeal to patients. According to the meta-analysis conducted by Rutkowska et al., the effects of VR training were lower than those of traditional PFMT, which remains the standard care for the treatment of UI. However, this paper only analyzed two papers. However, the results of this analysis can guide the design of future randomized controlled trials investigating the effect of RV-based PFMT in patients suffering from UI [32]. On the other hand, the study conducted by Elliott V. et al. showed that exercises performed with VR bring benefits in terms of reducing UI symptoms and also help to improve QOL, with patients showing a high level of satisfaction [56].

This innovative approach, which integrates dynamic functional and video gaming exercises, can enhance participants’ adherence to urinary incontinence treatment programs. However, further testing is required to confirm this potential.

This systematic review has certain limitations. Our research included six scientific databases, limiting the scope of our findings by excluding other databases (such as PubMed, Scopus, and others) that might have revealed additional studies. Furthermore, we did not apply inclusion or exclusion criteria based on outcome measures to examine as many articles as possible, resulting in significant heterogeneity.

## 5. Conclusions

The physical intervention represents a practical and efficacious therapeutic modality devoid of side effects, which reduces the severity of urinary incontinence, consequently enhancing patients’ overall quality of life.

Currently, there are a variety of physical approaches to managing OAB. Indeed, their number will continue to grow as the literature shows continuous concern regarding reducing OAB’s negative effects on patients’ quality of life.

The rehabilitation setting provides an excellent opportunity to promptly identify and start interventions for urinary incontinence. This is especially important due to the complex origins of neurogenic bladder dysfunction in individuals with neurological conditions. The presence of a multidisciplinary approach in inpatient rehab facilities is crucial for achieving or maintaining functional autonomy.

## Figures and Tables

**Table 1:** The quality of the studies included in the review.

Section	Girtner et al. [23]	Marzouk et al. [25]	Lucio et al. [29]	Gaspard et al. [26]	Stampas et al. [12]	Monteiro et al. [24]	Chen et al. [31]	Elmelund et al. [36]	Carilli et al. [37]	Perez et al. [38]	Ferreira et al. [39]
1	YES	YES	YES	YES	YES	YES	YES	YES	Yes	YES	YES
2	YES	YES	YES	YES	YES	YES	YES	YES	NO	YES	YES
3	-	-	NO	-	YES	-	-	YES	NO	YES	NO
4	YES	YES	YES	YES	YES	YES	YES	YES	YES	YES	YES
5	YES	YES	NO	-	YES	-	-	-	NO	-	NO
6	NO	-	NO	-	-	-	-	NO	NO	-	NO
7	NO	-	YES	YES	YES	-	-	YES	NO	-	NO
8	YES	YES	NO	NO	YES	YES	YES	NO	NO	NO	YES
9	YES	-	-	NO	YES	YES	NO	NO	YES	NO	NO
10	YES	YES	YES	YES	YES	YES	YES	YES	NO	YES	YES
11	YES	YES	YES	YES	YES	YES	YES	YES	YES	YES	YES
Total score	7/10	6/10	5/10	5/10	9/10	7/10	5/10	6/10	3/10	6/10	5/10

**Table 2:** Characteristics of the studies included in the review.

Study, Year, and Design	Population	Intervention/Comparison Supervision	Assessment Methods	Key Results
Gartner et al. [23], 2021	- **51 patients** (35% of all cases: MS) in 2 groups: - Group A = 25 - Group B = 26	- **Group A: TTNS**; biphasic rectangular pulses: 250 μs; impulse frequency of 20 Hz; impulse intensity: the individual pain threshold (exceeded 180 mA). - **Group B: TTNS**; biphasic rectangular: 250 μs; impulse frequency of 20 Hz; impulse intensity: the individual pain threshold (exceeded 180 mA); - Two urodynamic cycles and different electrode placement; - Each subject received simultaneous TTNS of tibial nerve for one urodynamic cycle and placebo stimulation during the other.	- I-QoL - ICIQ-SF 2004 - OABSS - Bladder Diary-3 days - Physical examination - Medical history symptoms - Cystometry.	- TTNS induced statistically significant improvements in bladder function compared to placebo stimulation. - Participants with anatomically normal lower urinary tracts were the only ones benefiting from TTNS. - TTNS could enhance deliberate bladder emptying by reinforcing voluntary detrusor contractions, leading to higher voiding volumes.
Marzouk et al. [25], 2022	- 40 patients—males RRMS in 2 groups: - CG = 20 - ESG = 20	- **CG group: PFMT** × 20 min; 3 times/week × 1 month - **ESG: PFMT** × 20 min and **PTNS** × 20 min-pulse: 250 ms; pulse frequency: 5 Hz; continuous mode (60 s on and 0 s off) 3 times/week × 1 month.	- OVBS - Cystometry - Abdominal ultrasound	- PTNS was considered a safe, effective, and superior to pelvic floor muscle training treatment option for neurogenic overactive bladder symptoms in people with MS.
Lucio et al. [29], 2016	- 30 patients—females Relapsing MS in 3 groups: - Group 1 = 10 - Group 2 = 10 - Group 3 = 10	- **Group 1: PFMT + EMG biofeedback and Sham Sacral NMES** × 30 min; 2 times/week × 12 weeks; - **Group 2: PFMT + EMG biofeedback + intravaginal NMES** × 30 min; 2 times/week × 12 weeks; - **Group 3: PFMT + EMG biofeedback + TTNS** × 30 min; 2 times/week × 12 weeks; * **PFMT**—50 ms pulse; frequency of 2 Hz with a stimulation time of 2 s, with a pause of 60 s between stimuli; * **SACRAL NMES**—50 ms pulse; frequency; 2 Hz; stimulation time of 2 s/60 s rest between stimuli; * **IVES NMES**—electrical pulses 200 μs; a frequency of 10 Hz; intensity tolerated by the participant; * **TTNS**—200 μs pulse; frequency of 10 Hz.	- 24 h pad test - Bladder diary-3 days - Pelvic floor muscle assessment - Cystometry - OAB-V8 - ICIQ-SF - Qualiveen Instrument	- A combination of PFMT and intravaginal NMES was more effective in decreasing PFM tone and improving flexibility and PFM relaxation after a maximal PFM contraction than PFMT alone or in combination with TTNS. - Treatment was also found to reduce the volume of urinary leakage measured via the 24 h pad test.
Gaspard et al. [26], 2014	- 31 patients—(16 males; 15 females) with MS in 2 groups: - Group 1 = 16 - Group 2 = 15	- **Group 1: PFMT with EMG biofeedback** (manometric anal, pelvic floor probe)—1 time/week × 30 min; - **Group 2: PTNS** rectangular biphasic current; a pulse duration of 220 μs; a frequency of 10 Hz—1 time/week × 30 min;	- “SF-Qualiveen” - Urinary Symptom Profile - Pelvic floor muscle assessment	- PTNS significantly improved the “overactive bladder” score and the frequency of daily voiding urges. - Patients treated with PFMT with EMG biofeedback report higher subjective satisfaction than patients in the STNTP group. This difference is not statistically significant.
Stampas et al. [12], 2019	- 19 patients diagnosed with SCI in: - Group 1 = 10 - Group 2 = 6	- **Group TTNS**—stimulation frequency: 10 Hz; pulse duration of 200 μs × 30 min/10 days (16-day period); st - **CG (Control Group) Sham stimulation**—stimulation frequency: 10 Hz; pulse duration of 200 μs × 30 min/10 days (16-day period).	- Cystometry	- TTNS is a safe and feasible modality treatment option for patients with acute SCI. - Sensation during bladder filling was significantly prolonged in the TTNS group.
Monteiro et al. [24], 2014	- 24 patients—males diagnosed with stroke into two groups: - Group 1 = 12 - Group 2 = 12	- **Treatment Group: PTNS**: frequency: 10 Hz; pulse duration of 200 ms continuously; ×30 min; 2 times/week × 6 weeks; - **CG (Control Group)**: 12 sessions of **stretching** of the lower limb muscles; 3 series of stretches of 30 s each at home.	- Bladder diary - Personal and family history - The Barthel index	- No differences between patients in the ES and placebo groups regarding the demographic variables used for assessing between-group homogeneity. - ES of the posterior tibialis nerve is considered an effective and safe treatment method for NOB and is not associated with side effects.
Chen et al. [31], 2015	- 100 patients with SCI in 2 groups: - Group 1 = 50 - Group 2 = 50	- **Group A: PtNS**–continuous bipolar square-wave electrostimulation therapy; pulse duration: 200 μs; stimulation frequency: 20 Hz; intensity: 24–26 mA; ×30 min; 2 times/week × 4 weeks; - **Group B**: received treatment with Solifenacin Succinate × 4 weeks.	- Bladder diary-3 days - Personal and family history - I-QOL - Physical examination - Urinary tract ultrasound	- Only in group B adverse effects were recorded, so two patients withdrew due to side effects. - no significant differences have been reported between the two types of therapy. - PtNS therapy is noninvasive, and no adverse effects have been reported.
Elmelund et al. [36], 2017	- 36 patients—females diagnosed with SCI in 2 groups: - Group 1 = 17 - Group 2 = 19	- **Group A: PFMT** (30 maximal contractions × 5–10 s/10 s pause); - **Group B: PFMT** (30 maximal contractions × 5–10 s/10 s pause) + **IVES** (intermittent stimulation-frequency: 40 Hz; pulse duration: 250 μs-for 7.5–10 min; 30 stimulation cycles; 5–10 s of stimulation/10 s pauses and continuous stimulation: frequency: 10 Hz; pulse duration: 250 μs; for 10–20 min; - Both groups: daily × 12 weeks.	- Digital vaginal and rectal palpation - EMG Biofeedback - ICIQ-UI-SF - Cystometry - Pressure-flow study	- After a 12-week training period, only the PFMT group showed significant improvement in the ICIQ-UI-SF score and daily urinary incontinence episodes. - PFMT should be recommended as first-line conservative treatment of UTI in women with incomplete SCI.
Carilli et al. [37], 2023	- 33 patients—26 females; - 7 males diagnosed with MS:	- **PtNS**—frequency of 20 Hz; pulse width of 200 ms, the stimulation current (0–10 mA); current intensity: increased based on patient’s tolerability; - 12 stimulation sessions × 30 min/twice weekly × 6 week.	- Bladder diary-3 days - IPSS - ICIQ-SF - OABQ-SF	- After 12 sessions of PtNS, most patients (72.4%) reported a significant reduction in the frequency, urgency, and urge of incontinence episodes in their bladder diaries.
Perez et al. [38]	- 48 patients diagnosed with RRMS: - CG = 21 - EG = 19	- **CG**: 8–12 slow contractions, then an additional 3–4 rapid contractions, three times daily for ×12 weeks, home-based; - **EG**: PFMT with the help of internal palpation for 30 min weekly with a physiotherapist × 12 weeks, supervised + 8–12 slow contractions, then an additional 3–4 rapid contractions, three times daily × 12 weeks, home-based.	- Bladder diary-3 days - ICIQ-SF - OABQ-SF - Exercise diary	- Physiotherapist-guided PFMT was linked to a decrease in the number of leakages in both men and women. - The guided PFMT group tended to have better adherence to treatment (89% compared to 77% for the unguided group.
Ferreira et al. [39]	- 30 patients (females) diagnosed with MS: - CG = 15 - EG = 15	- **CG: PMFT**: 3 sets of 8–10 contractions, twice a week × 6 months, home-based; - **EG: PMFT**: 3 sets of 8–10 contractions + 20 fast and 20 slow contractions with IVES of 30 min, twice a week × 6 months, supervised.	- HRQOL overactive bladder severity follow-up	- Pelvic floor muscle strengthening exercises have proven beneficial in improving lower urinary tract symptoms and quality of life. - Results have shown that electrostimulation and pelvic floor strengthening training enhance the outcomes.

* PFMT: pelvic floor muscle training; * SACRAL NMES: sham neuromuscular electrical stimulation placed over the sacrum; * IVES NMES: intravaginal neuromuscular electrical stimulation; * TTNS: transcutaneous tibial nerve stimulation.

## Supplementary Materials

The following supporting information can be downloaded at: https://www.mdpi.com/article/10.3390/medicina60071152/s1.
